# Gelatine Suppositories

**Published:** 1879-12

**Authors:** 


### ARTICLE XI.

### GELATINE SUPPOSITORIES.

MESSRS. EDITORS: — Permit me to enquire through your pages whether physicians have often obtained satisfactory results from the use of suppositories prepared with gelatine. In two cases where I used them, they failed to dissolve. In one of these cases the suppository remained in the rectum one hour, and in the other all night, without any appreciable alteration in size and form. E. F. I.

---

DR. E. S. GAILLARD, editor of the *Richmond and Louisville Medical Journal* and of the *American Medical Bi-Weekly*, has removed to New York, and taken his journals with him.

